# Molecular evolution of a gene cluster of serine proteases expressed in the *Anopheles gambiae *female reproductive tract

**DOI:** 10.1186/1471-2148-11-72

**Published:** 2011-03-19

**Authors:** Emiliano Mancini, Federica Tammaro, Francesco Baldini, Allegra Via, Domenico Raimondo, Phillip George, Paolo Audisio, Igor V Sharakhov, Anna Tramontano, Flaminia Catteruccia, Alessandra della Torre

**Affiliations:** 1 Istituto-Pasteur - Fondazione Cenci Bolognetti, Dipartimento di Sanità Pubblica e Malattie Infettive, 'Sapienza' Università di Roma, Rome, Italy; 2 Dipartimento di Medicina Sperimentale e Scienze Biochimiche, Università di Perugia, Terni, Italy; 3 Dipartimento di Scienze Biochimiche, 'Sapienza' Università di Roma, Rome, Italy; 4 Department of Entomology, Virginia Tech, Blacksburg, VA, USA; 5 Dipartimento di Biologia e Biotecnologie "C. Darwin", 'Sapienza' Università di Roma, Rome, Italy; 6 Division of Cell and Molecular Biology, Imperial College London, London, UK

**Keywords:** molecular evolution, reproduction, adaptive evolution, gene duplication, *Anopheles gambiae *complex

## Abstract

**Background:**

Genes involved in post-mating processes of multiple mating organisms are known to evolve rapidly due to coevolution driven by sexual conflict among male-female interacting proteins. In the malaria mosquito *Anopheles gambiae *- a monandrous species in which sexual conflict is expected to be absent or minimal - recent data strongly suggest that proteolytic enzymes specifically expressed in the female lower reproductive tissues are involved in the processing of male products transferred to females during mating. In order to better understand the role of selective forces underlying the evolution of proteins involved in post-mating responses, we analysed a cluster of genes encoding for three serine proteases that are down-regulated after mating, two of which specifically expressed in the atrium and one in the spermatheca of *A. gambiae *females.

**Results:**

The analysis of polymorphisms and divergence of these female-expressed proteases in closely related species of the *A. gambiae *complex revealed a high level of replacement polymorphisms consistent with relaxed evolutionary constraints of duplicated genes, allowing to rapidly fix novel replacements to perform new or more specific functions. Adaptive evolution was detected in several codons of the 3 genes and hints of episodic selection were also found. In addition, the structural modelling of these proteases highlighted some important differences in their substrate specificity, and provided evidence that a number of sites evolving under selective pressures lie relatively close to the catalytic triad and/or on the edge of the specificity pocket, known to be involved in substrate recognition or binding. The observed patterns suggest that these proteases may interact with factors transferred by males during mating (e.g. substrates, inhibitors or pathogens) and that they may have differently evolved in independent *A. gambiae *lineages.

**Conclusions:**

Our results - also examined in light of constraints in the application of selection-inference methods to the closely related species of the *A. gambiae *complex - reveal an unexpectedly intricate evolutionary scenario. Further experimental analyses are needed to investigate the biological functions of these genes in order to better interpret their molecular evolution and to assess whether they represent possible targets for limiting the fertility of *Anopheles *mosquitoes in malaria vector control strategies.

## Background

Sexual reproduction in organisms with internal fertilization is known to be mediated by a series of molecular interactions between the male ejaculate and female reproductive factors [1,2]. Since these interactions are fundamental to fertilization and, thus, for organismal fitness, molecular coevolution has been suggested to arise between male components and interacting female proteins [1,3,4]. While rapid evolution driven by positive selection has been extensively documented in reproductive proteins from a number of organisms in which multiple matings occur (e.g. in *Drosophila *sp.), studies in monandrous species have been so far neglected, as it has been argued that low divergence levels should be expected because of the reduced extent of sexual selection (e.g. cryptic female choice of male traits) and/or sexual conflict (i.e. the evolutionary arms race between the sexes, where each sexual counterpart attempts to achieve its own reproductive optimum at a fitness cost to the opposite sex) [5,6].

In *Anopheles gambiae *sensu stricto (s.s.), the major malaria vector species in Sub-Saharan Africa, females mate a single time during their lifetime, after which they become refractory to further copulation. Multiple matings in natural populations of *A. gambiae *s.s. occur in a small percentage of individuals (~2%) and may be caused by the incomplete transfer of male seminal secretions [7,8]. This species belongs to the *A. gambiae *complex which includes other six morphologically indistinguishable allied species and two incipient M and S molecular forms within *A. gambiae *s.s. [9-11]. Only few data on the frequency of multiple matings in natural populations are available for these closely related taxa of *A. gambiae *s.s. (e.g. 0.13% in *A. melas*) [12], however, in general, females of the *A. gambiae *species complex are believed to be monandrous. Although the molecular triggers of female refractoriness to multiple copulations are not yet known, it has been observed that transfer of male seminal secretions is essential for modulating *A. gambiae *s.s. female post-mating physiology and behavior [8,13]. Seminal secretions produced in the male accessory glands (MAGs) are transferred to the female atrium (uterus) during copulation in the form of a gelatinous 'mating plug', which is digested within 24 hours after copulation [12]. Recent studies showed that the mating plug is not an efficient physical barrier to re-insemination, but it is an essential reproductive feature in *A. gambiae *s.s., as its formation and transfer are necessary to ensure correct sperm storage by the female [8]. Moreover, recent data strongly points to a key role of atrial proteolytic enzymes in plug digestion: in fact i) these proteases are expressed at high levels in the virgin atrium and considerably down-regulated by 24 hours after mating [14] when the mating plug is mostly digested, and ii) some of them were detected by mass spectrometry analysis on mating plug samples dissected from freshly mated females [8].

**Figure 1:**
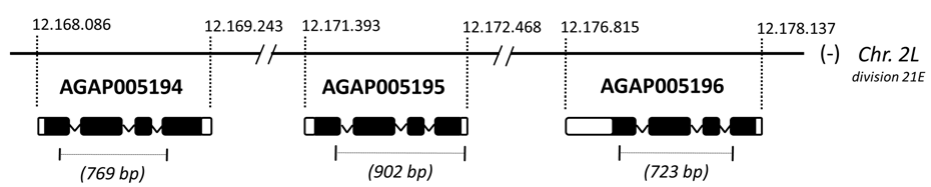
**Location of sequenced fragments of the three serine protease genes on *Anopheles gambiae *genome**. Fragment lengths are in parentheses. The three genes are located on minus strand of chromosome 2L, division 21E. Numbers above the line indicate the coordinates on the genome map (*A. gambiae *PEST genome ver. 3.5, Sept. 2009).

In this study we examined the patterns of molecular evolution of three female-expressed serine proteases that are encoded by three genes (namely AGAP005194, AGAP005195 and AGAP005196) clustered on chromosome 2L in the *A. gambiae *genome (Figure 1). These are amongst the *A. gambiae *s.s. genes most strongly down-regulated by mating: AGAP005194 and AGAP005195 are exclusively expressed in the atrium and are associated with the mating plug [8,14], whereas AGAP005196 is predominantly expressed in the sperm storage organ, the spermatheca [14]. These proteolytic enzymes may therefore play a role in mating plug digestion and/or other reproductive processes important for mosquito fertility.

Our main interest was to highlight signatures of adaptive evolution in these 3 serine protease genes within the well-defined cryptic and incipient (i.e. the M and S forms) species of the *A. gambiae *complex. In fact, there is a considerable intrinsic value in studying the role of natural selection in genes controlling reproduction in the most important malaria vectors in Sub-Saharan Africa, as a better knowledge of the still largely unknown genetic bases of their post-mating physiological and behavioral responses could open perspectives for the development of novel tools to manipulate their fertility and fecundity. In addition, the recently radiated *A. gambiae *species represent an interesting model to study the adaptive evolution of genes potentially involved in the build-up of reproductive isolating barriers. However, the application of selection-inference methods in closely related taxa, such as those of the *A. gambiae *complex, imposes some limitations and a critical evaluation of the results [15,16]. Thus, to provide the best possible interpretation of the inferred positive selected sites and to corroborate their possible functional significance, these were mapped on the reconstructed 3D models of the three serine proteases.

Overall, our results stimulate further studies to elucidate the role of this gene family in determining the reproductive success of *A. gambiae *taxa and allow to speculate on the relative importance of selective forces underlying the evolution of post-mating characters by comparing the observed patterns with those available for polyandrous organisms.

## Results

### Sequencing data

Partial sequences of 489, 603 and 456 bp were obtained for the coding regions of AGAP005194 (transcript = 940 bp, total protein = 272 aa), AGAP005195 (transcript = 867 bp, total protein = 250 aa) and AGAP005196 (transcript = 1115 bp, total protein = 264 aa), respectively (Figure 1). Furthermore, under the same PCR conditions used for the amplification of AGAP005196, we identified a novel paralog, not yet annotated in the *A. gambiae *genome. This paralog is similar to AGAP005196 but characterised by a 368 bp insertion of a miniature inverted repeat transposable element (MITE) in the third intron (Additional file 1). This inserted element is 61.8% AT-rich and forms a putative stable secondary structure, as characteristic of the TA-I-α-Ag MITE family [17]. Because of these features, the MITE insertion represented a difficult template for amplification and, despite several efforts, we failed to optimize PCR/sequencing protocols. However, we were able to obtain 9 sequences for 4 out of the 5 analysed species of the *A. gambiae *complex. FISH experiments (Additional file 2) performed on *A. gambiae*, *A. arabiensis *and *A. merus *chromosomes using a probe binding to the common sequences (intron 2 and exon 3) of both AGAP005196 and the novel copy revealed a single signal in subdivision 21E of the 2L chromosome arm. The same result was obtained using an additional probe. Overall, these results suggest that the novel paralog is placed in the same chromosomal division (2L, 21E) of the other three genes (and possibly in the same gene cluster, Figure 1) in *A. gambiae *and in all examined species of the *A. gambiae *complex, indicating that a tandem gene duplication occurred in this specific genomic region.

### Divergence, polymorphisms and gene tree inferences

**Figure 2:**
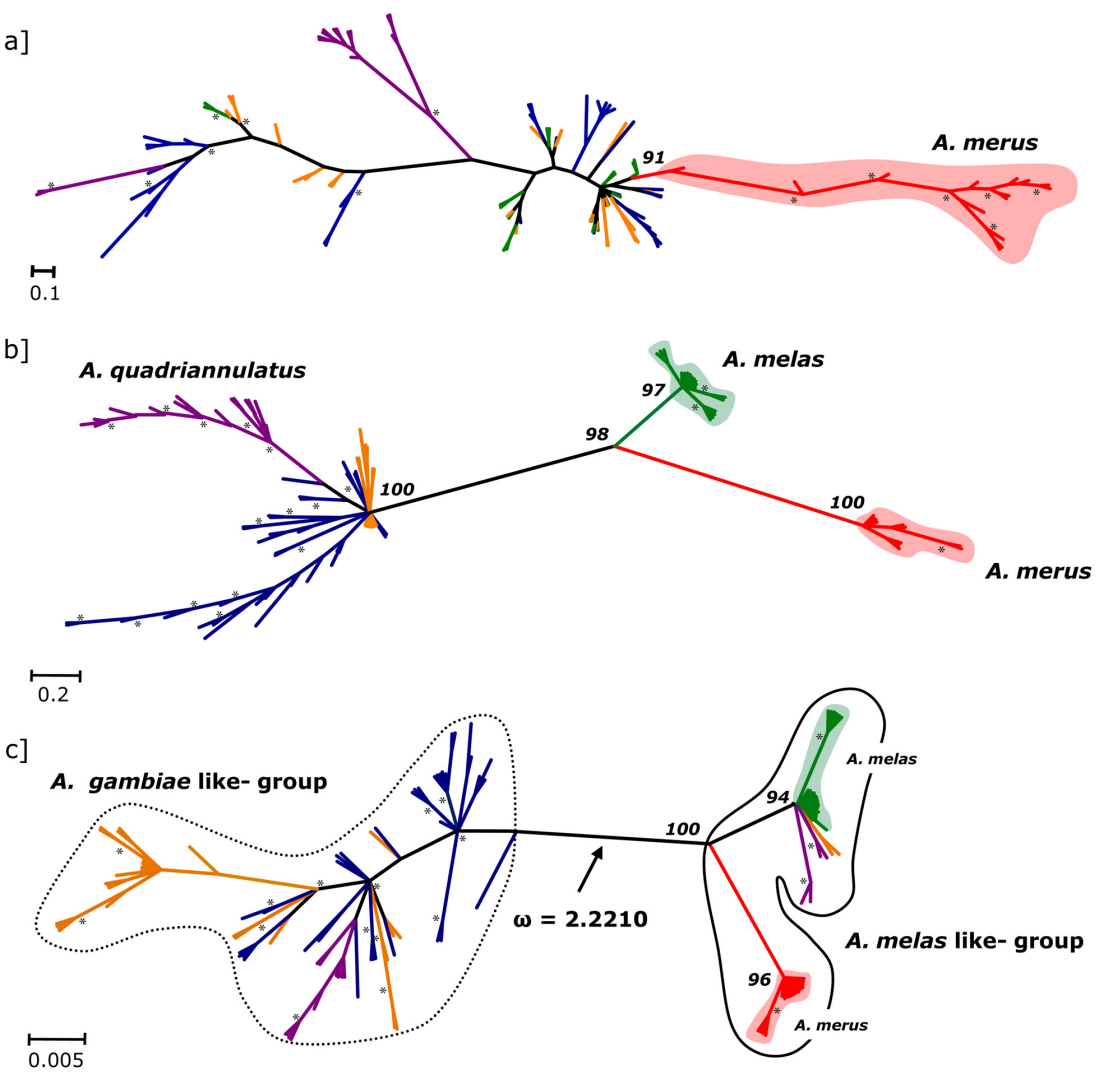
**50% majority-rule consensus bayesian (unrooted) trees of a) AGAP005194, b) AGAP005195, c) AGAP005196**. Posterior probabilities of clades discussed in the text are reported above nodes. Nodes supported by a posterior probabilities ≥ 0.95 are indicated by *. Branches leading to single individuals (or included in specific-lineages) are depicted with species-specific colours: *A. gambiae *(blue), *A. arabiensis *(yellow), *A. quadriannulatus *(violet), *A. melas *(green), *A. merus *(red); monophyletic clades are shaded accordingly. The value of ω (> 1) in AGAP005196 is reported below the branch separating the *A. gambiae*-like from the *A. melas*-like groups of alleles (enclosed in dashed and contiguous lines, respectively). In all trees, branch lengths are scaled according to nucleotide substitutions per site.

Divergence (and polymorphisms) among the species of the *A. gambiae *complex are reported below for each gene and summarized in Table 1 (and Additional file 3). Bayesian gene trees reconstructed from the coding regions of each gene are depicted in Figure 2. In general, we found that *A. merus *and *A. melas *were more frequently included in monophyletic clusters, whereas *A. gambiae *M- and S- molecular forms (undergoing a process of incipient speciation [10]) shared many alleles at all loci. Henceforth *A. gambiae *s.s. was considered as a single taxonomic unit in some of our subsequent analyses. The results obtained are reported below for each gene analysed.

**Table 1:** McDonald-Kreitman (MK) tests and genetic divergence

	**AGAP005194**						**AGAP005195**						**AGAP005196**					
	
	**Fixed**		**Polym**.				**Fixed**		**Polym**.				**Fixed**		**Polym**.			
									
	**S**	**NS**	**S**	**NS**	***p***	**Dxy**	**S**	**NS**	**S**	**NS**	***p***	**Dxy**	**S**	**NS**	**S**	**NS**	***p***	**Dxy**
	
**ga-ar**	0	0	26	29	n.s.	0.027	0	0	15	22	n.s.	0.010	0	0	18	29	n.s.	0.023
**ga-qd**	0	0	26	35	n.s.	0.049	0	0	15	24	n.s.	0.017	0	0	16	25	n.s.	0.019
**ga-ml**	0	0	25	24	n.s.	0.022	8	13	15	21	1.000	0.046	**0**	**8**	**15**	**17**	0.016^*^	0.037
**ga-mr**	1	2	26	35	1.000	0.059	9	22	13	24	0.615	0.067	4	5	14	15	1.000	0.038
**ar-qd**	1	0	19	36	0.357	0.049	1	1	6	10	1.000	0.014	0	0	11	25	n.s.	0.026
**ar-ml**	0	0	14	23	n.s.	0.022	8	13	4	5	1.000	0.039	0	0	10	24	n.s.	0.035
**ar-mr**	1	2	17	35	1.000	0.058	9	23	2	8	0.705	0.060	4	2	9	21	0.161	0.044
**qd-ml**	1	0	17	29	0.383	0.046	8	14	6	7	0.724	0.046	0	0	7	18	n.s.	0.030
**qd-mr**	2	2	17	35	0.598	0.059	8	23	4	10	1.000	0.064	4	2	6	16	0.147	0.030
**ml-mr**	1	2	15	28	1.000	0.053	7	15	2	5	1.000	0.040	5	4	1	4	0.301	0.023

S = synonymous sites, NS = nonsynonymous sites; *p-*values computed by Fisher's exact test; * significant *p*-value

(< 0.05) for MK test; ga = *A. gambiae*, ar = *A. arabiensis*, qd = *A. quadriannulatus*, ml = *A. melas*, mr = *A. merus*

AGAP005194: on average, 80 segregating sites were found in the coding region of this gene (16% of the total number of nucleotide sites) and 41 out 163 (25%) amino acid positions were variable. The average nucleotide diversity (π) was 0.036. Out of the 102 sequences obtained, 57 different alleles were found. The highest haplotype diversity (Hd) was found in *A. gambiae *S-form (0.980) and in *A. arabiensis *(0.965), whereas the lowest value was detected in *A. quadriannulatus *(0.582). π within species/forms ranged from 0.014 (*A. melas*) to 0.028 (*A. gambiae *S-form) and from 0.017 to 0.041 and from 0.011 to 0.025 at synonymous (π_s_) and nonsynonymous (π_a_) sites, respectively (Additional file 3). Dxy ranged from 0.022 to 0.059, with the highest values of divergence found in pairwise comparisons with *A. merus *(Table 1). The phylogenetic tree based on the HKY+I+G (pinv = 0.6580; shape = 0.6680) model was not fully resolved (Figure 2a): most species were included in non-monophyletic assemblages (gsi values = 0.25-0.56), with the exception of *A. merus *(gsi = 1.0). This species clustered in a separated clade (supported by a posterior probability of 0.91), although embedded in a larger clade also including individuals from other species.

AGAP005195: a total of 80 segregating sites were found in the coding region of this gene (13% of the total number of nucleotide sites), and 43 out 201 (21%) amino acid sites were variable. The average π was 0.029. Out of the 96 allele sequences obtained, 53 haplotypes were found. The highest and the lowest Hd values were found in *A. gambiae *(0.986) and *A. melas *(0.699), respectively. Species/forms π ranged from 0.001 (*A. melas*) to 0.011 (*A. gambiae*). A null value of π_s _was found in *A. merus *(i.e. absence of intra-specific synonymous substitutions), whereas the highest value π_s _was observed in *A. gambiae *M-form (0.023). The lowest value of π_a _was scored in *A. melas *(0.001) and the highest in *A. gambiae *(0.009) (Additional file 3). Dxy ranged from 0.010 (*A. gambiae *vs. *A. arabiensis*) to 0.067 (*A. gambiae *vs. *A. merus*) (Table 1). In the phylogenetic tree based on the HKY+I+G model (pinvar = 0.6830; shape = 0,7280), alleles of *A. melas *and *A. merus *are grouped in two monophyletic clades (supported by posterior probability values of 0.97 and 1.0, gsi = 0.93 and 1.0, respectively) that are clustered together (0.98 support). *A. quadriannulatus *alleles are also grouped together in a single clade (1.0 support; gsi = 1.0), but embedded in a larger clade where *A. gambiae *and *A. arabiensis *alleles are mixed.

AGAP005196: a total of 58 segregating sites were found in the coding region in this gene (13% of the total number of nucleotide sites), and 34 out 152 (22%) amino acid sites were variable. The average π was 0.026. Out of the 122 allele sequences, 49 haplotypes were found. The highest and lowest Hd values were found in *A. gambiae *(0.960) and *A. merus *(0.400), respectively. Species/forms π ranged from 0.001 (*A. merus*) to 0.018 (*A. quadriannulatus*); π_s _ranged from a null value (*A. merus*) to 0.028 (*A. arabiensis*) and π_a _from 0.001 (*A. merus*) to 0.017 (*A. quadriannulatus*) (Additional file 3). Dxy ranged from 0.019 (*A. gambiae *vs. *A. quadriannulatus*) to 0.044 (*A. arabiensis *vs. *A. merus*). In the bayesian phylogenetic reconstruction based on the HKY+I (pinvar = 0.8170) model, two major groups were strongly separated and supported at their nodes: one group (hereafter named as the *A. gambiae*-like group) includes all *A. gambiae *alleles and most of *A. arabiensis *+ *A. quadriannulatus *alleles. The second clade (hereafter named as the *A. melas*-like group) includes all alleles of *A. melas *and *A. merus*, 2 *A. arabiensis *alleles (from 1 Kenyan specimen) and 4 *A. quadriannulatus *alleles (from 2 Zimbabwean specimens). In particular, *A. merus *(gsi = 1.0) is well separated from *A. melas *(with a high level of exclusive ancestry of its alleles, gsi = 0.86), and alleles of *A. arabiensis *and *A. quadriannulatus *are included in the same clade. In the *A. gambiae*-like group, on the contrary, alleles are not split in species-specific monophyletic groups, although *A. gambiae *alleles show a quite high level of exclusive ancestry (gsi = 0.71).

### *d*_*N*_/*d*_*S *_pairwise comparison and McDonald-Kreitman test

**Figure 3:**
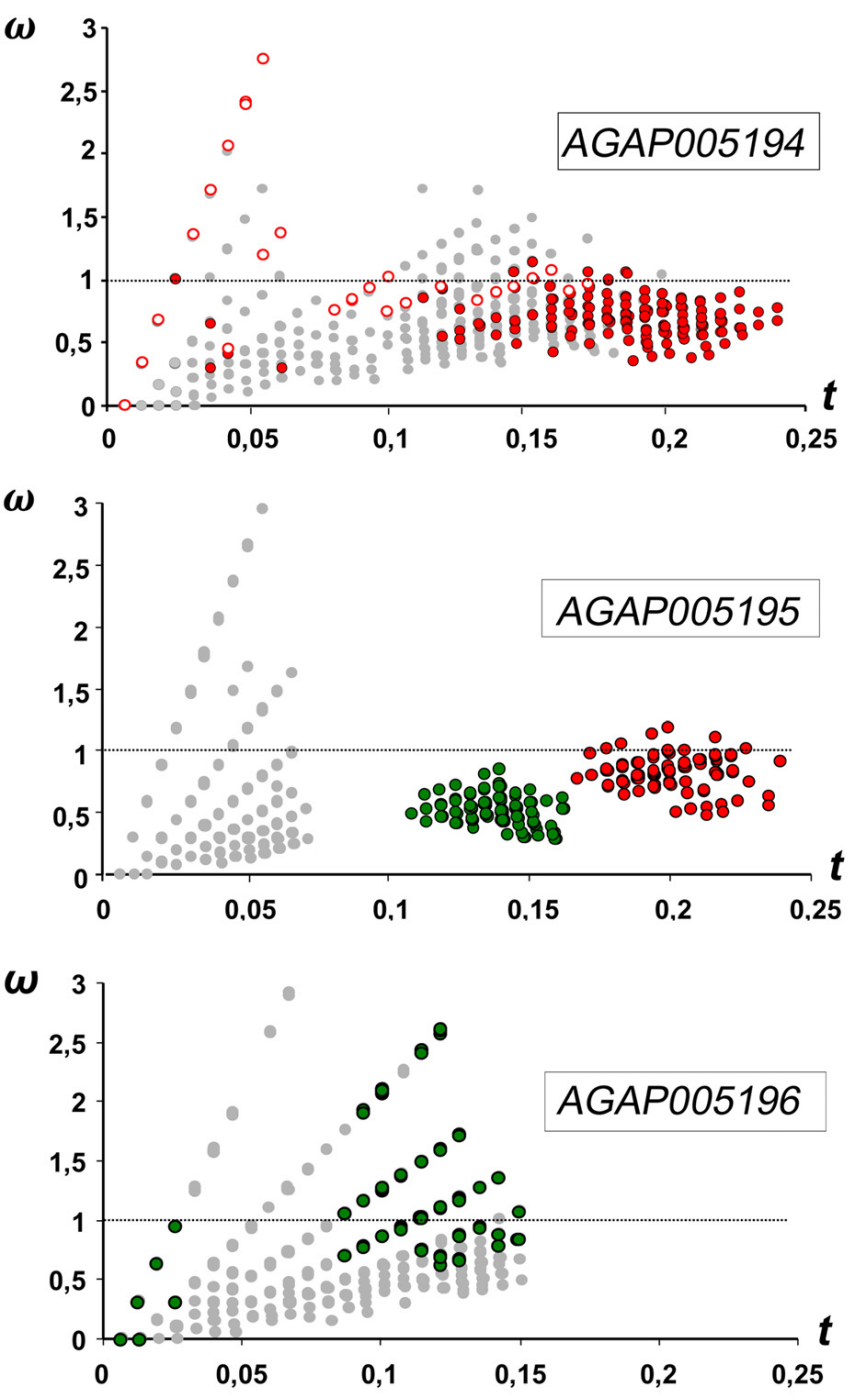
**Pairwise maximum likelihood estimates of ω (= *d***_***N***_**/*d***_***S***_**) plotted against the estimates of sequence divergence (*t*)**. Red- and green-filled circles represents inter-specific comparisons with *A. merus *and *A. melas*, respectively. *A. merus *intra-specific comparisons are represented with red-open circles. The straight line indicates the neutral expectation (ω = 1)

Pairwise comparisons of Maximum Likelihood (ML) estimates [18] of *d*_*S *_and *d*_*N *_are plotted in Figure 3. The range of sequence divergence - defined as the expected number of nucleotide substitutions per codon (*t*) - is comparable for AGAP005194 and AGAP005195 (0.00-0.24), whereas a smaller *t *range was scored for AGAP005196 (*t *= 0.00-0.15). In general, a pattern of puryfing selection was observed for AGAP005194, especially at high divergence levels. However, most of *A. merus *intra-specific comparisons for *t *< 0.06 showed ω > > 1 (or 'infinite'; i.e. absence of intra-specific synonymous polymorphisms), whereas ω was ~ 1 at 0.08 <*t *< 0.17. In addition, at *t *< 0.05, ω > > 1 was also scored for several intra-specific comparisons within the *A. gambiae *S-form and *A. arabiensis*, and for some inter-specific comparisons. A general pattern of purifying selection (*d*_*S *_>*d*_*N*_) was also observed for AGAP005195. Apart from values scored at a low divergence level (*t *< 0.1), the other pairwise comparisons fell in two discrete and strikingly delimitated clusters corresponding to: i) all inter-specific comparisons with *A. melas *(*t *= 0.11-0.16) and, ii) all inter-specific comparisons with *A. merus*, except for those with *A. melas *that are already included in the first cluster (*t *= 0.17-0.24). Since the computed genetic distances are not independent from the data used to estimate *d*_*N *_and *d*_*S*_, it is notable that divergence estimates in most inter-specific pairwise comparisons involving *A. merus *are inflated for the co-occurence of a high number of replacements and the absence of synonymous substitutions at intra-specific level. In fact, the relationship between *d*_*S *_and genetic distances is linear only when *A. merus *is not considered (r^2 ^= 0.9207, slope = 0.5392), whereas linearity for *d*_*N *_and genetic distances is observed even when computed on the whole dataset (r^2 ^= 0.9745, slope = 0.3047). Also for this gene, ω is > > 1 and increases linearly with different slopes in pairwise comparisons at divergence levels < 0.1. As already mentioned, a smaller *t *range was scored for AGAP005196 (*t *= 0.00-0.15). It is worth to note that ω increases steadily with *t *in this gene - following a similar trend to that observed for the other two genes in the same range of divergence - and that ω was > 1 (1.07-5.26) in most of inter-specific comparisons involving *A. melas*.

This relative preponderance of nonsynonymous changes has been also noted in closely related sequences obtained from other taxa [19,20]. As argued by some authors, ω estimates from a set of conspecific sequences are not appropriate to detect patterns of selection, because the observed differences at this level represent segregating polymorphisms as opposed to fixed substitutions [21]. This consideration should be also extended to closely related species, whose introgression and/or recent ancestry affect lineage sorting of alleles, so that differences among them might not represent fixation events along independent lineages. This implies that it might be difficult to detect adaptive evolution in a relatively short time after radiation of closely related species, and that fixation of species-specific replacements is likely to be achieved only under strong positive selection [22]. Because of these limits, for each positive selected site detected by ML approaches (see below) the state of character was carefully examined along the branches of the gene-trees: caution was taken in interpreting the inflated ω at some of these sites solely as an effect of a long-term change in selective pressures, because shared mutations more likely represented ancestral replacement polymorphisms rather than multiple independent substitutions.

Results of McDonald-Kreitman tests are shown in Table 1. For all genes, the number of replacements exceeded the number of synonymous substitutions at polymorphic sites in almost all pairwise comparisons, although this difference was not significant. The number of fixed synonymous and nonsynonymous substitutions among species was very low for AGAP005194 and AGAP005196, whereas in AGAP005195 a relatively high number of fixed replacements was observed in pairwise comparisons involving *A. merus *and *A. melas*. A significant *p*-value of MK test (*p *< 0.05) was only found for AGAP005196 between *A. gambiae *and *A. melas*.

### Recombination detection

The analysis using the GARD algorithm and the RDP software did not detect any statistically significant recombinant, or gene conversion among the three serine protease genes.

### Selection tests using ML approaches (PAML and HyPhy)

For AGAP005194 and AGAP005195, likelihood ratio tests were significant for both model comparisons (M2 *vs. *M1 and M8 *vs. *M7) highlighting several sites with ω values higher than 1. These positively selected sites were identified by both NEB and BEB analyses (posterior probability = 0.99) (Table 2). For AGAP005194, four residues (i.e. 42, 43, 121, 161) were consistently identified by NEB and BEB of M8 and M2 models. The ω ratios at these sites were > 1 (ω~5) in all cases, even when considering the standard errors (S.E.) of estimates. All HyPhy analyses indicated that site 42 is evolving under positive selection; FEL also identified site 5, while the results of REL were in accordance with those of NEB and BEB. For AGAP005195 seven residues (i.e. 51, 93, 141, 157, 186, 199, 201) were identified by BEB and NEB of M2 and M8 models. The ω ratios at these sites were ~8-9 in all cases, also when considering the S.E. of estimates. FEL, SLAC and REL identified residues 186, 199 and 201 as evolving under positive selection. For AGAP005196, none of the comparisons among codon models was significant using PAML. SLAC did not identify sites evolving under positive selection in this gene, whereas FEL identified sites 67 and 109. Finally, the less conservative REL method identified 13 positively selected sites for this gene: 8, 16, 18, 37, 39, 59, 67, 81, 82, 94, 109, 113, 124.

**Table 2:** Site-by-site detection of positive selection (PAML and HyPhy)

	AGAP005194					AGAP005195					AGAP005196				
	
Site model	**ω**^**a**^	**p (ω)**^**b**^	lnL	**χ**^**2**^	*p*	**ω**^**a**^	**p (ω)**^**b**^	lnL	**χ**^**2**^	*p*	**ω**^**a**^	**p (ω)**^**b**^	lnL	**χ**^**2**^	*p*
**M1a *(nearly neutral)***	1.00	0.32	-1732.78	34.97	**1 × 10^-6^**	1.00	0.26	-1810.86	69.72	**0.00**	1.00	0.27	-1319.28	4.04	0.14
**M2a *(selection)***	5.42	0.04	-1715.29			9.23	0.04	-1776.00			3.91	0.03	-1317.26		

**M7 *(beta)***	1.00	0.10	-1732.88	35.11	**1 × 10**^**-6**^	1.00	0.10	-1811.09	70.12	**0.00**	1.00	0.10	-1319.60	4.76	0.09
**M8 *(beta & ω)***	5.22	0.05	-1715.32			9.12	0.04	-1776.03			3.55	0.05	-1317.22		

**Sites with *d***_***N***_***/d***_***S***_**> 1**	5^c^, **42**, 43^d^, 48, 74, 121^d^, 161^d^					37^e^, 51, 93, 141, 157, **186**, **199**, **201**					8^d^, 16^d^, 18^d^, 37^d^, 39^d^, 59^d^, 67^d^, 81^d^, 82^d^, 94^d^, 109^d^, 113^d^, 124^d^				

^a ^Estimate of the highest ω value for any codon, ^b ^Proportion of codons with the highest ω value

Residues identified by FEL^c ^, REL^d^, and BEB from M2^e ^only; residues identified by all analyses (PAML and HyPhy) are in bold

The branch model tests applied to AGAP005194 dataset did not support episodic selection along any branch, whereas it suggested a putative long-term positive selection scenario in *A. merus*, although the averaged ω value assigned to foreground branches (ω = 1.09) was not significantly > 1 (Table 3). In AGAP005195, we also tested for alternative hypotheses of selection (H_1 _and H_2_) in *A. merus *and *A. melas*. The branch model applied to branches leading to *A. melas *and *A. merus*, respectively, did not support positive selection. A long-term selection scenario, although not significant, showed a better likelihood score (-1857.9950) with a mean ω = 1.38 for the foreground branches of the *A. merus *clade. However, the branch-site test was highly significant (using a *p*-value adjusted after Bonferroni's correction, Table 3) when applied to the branch leading to the clade grouping *A. melas *and *A. merus*, and BEB identified one positively selected site (residue 71). In AGAP005196 the branch model detected episodic selection along the branch separating the *A. melas*-like from the *A. gambiae*-like groups (ω = 2.22, p < 0.05, see Figure 2c). The branch model performed using a gene-tree reconstructed after excluding *A. arabiensis *and *A. quadriannulatus *from the dataset also detected episodic selection for the branch separating *A. gambiae *from the other two species (ω = 2.14; p < 0.05). However, in both cases, ω was not significantly > 1. The high ω along the branch separating the *A. gambiae*- and *A. melas*- like groups suggests that positive selection has been acting on it; however, the *p*-value < 0.05 cannot be directly interpreted as significant due to a lack of *a priori *specification of the foreground branches. As already pointed out above, this result is probably affected by an overestimation of ω due to the presence of several segregating replacements. In fact, although the presence of four fixed replacements between the two groups (and the absence of fixed synonymous substitutions along this branch) is consistent with the hypothesis of positive selection, other non-synonymous changes are shared at 10 out 34 sites (29%; synonymous changes at 2 out 24 sites, 8%) among the species/individuals belonging to the two clusters on both sides of the branch. The most parsimonious interpretation is that such replacements represent ancestral polymorphisms rather than multiple independent substitutions in different lineages, and this likely affects the reliability of ω estimates.

**Table 3:** Branch- and branch-site detection of positive selection (PAML)

Model	**ω **_**(back)**_	**ω **_**(fore)**_	lnL	**χ**^**2**^	*p*	Model	**ω **_**(back)**_	**ω**_**(fore)**_	lnL	**χ**^**2**^	*p*
**AGAP005194 - branch test (foreground ω> background ω)**						**AGAP005196 - branch test (foreground ω> background ω)**					

**Test for *A. merus***						**Test for *A. melas *+ *A. merus***					
H0	0.46	0.46	-1779.96			H0	0.34	0.34	-1331.85		
H1	0.45	**infinite**	-1780.10	n.a.	n.a.	H1	0.31	**2.22**	-1329.46	4.77	**0.03**
H2	0.36	**1.09**	-1776.10	7.72	**0.01**	H2	0.27	0.66	-1329.97	3.76	0.05

**AGAP005194 - branch test (foreground ω> 1)**						**AGAP005196 - branch test (foreground ω> 1)**					

H0 (ω = 1)	0.36	1.00	-1776.13			H0 (ω = 1)	0.31	1.00	-1329.79		
H2	0.36	**1.09**	-1776.10	0.05	0.82	H1	0.31	**2.22**	-1329.46	0.66	0.42

**AGAP005195 - branch test (foreground ω> background ω)**						**AGAP005196**^**§ **^**- branch test (foreground ω> background ω)**					

**Test for *A. merus***						**Test for *A. melas***					
H0	0.62	0.62	-1859.32			H0	0.38	0.38	-1067.31		
H1	0.59	0.97	-1858.97	n.a.	n.a.	H1	0.35	**infinite**	-1065.96	2.70	1.00
H2	0.55	1.38	-1858.00	2.65	0.10	H2	0.33	**1.59**	-1066.02	2.58	0.11
**Test for *A. melas***						**Test for *A. melas *+ *A. merus***					
H0	0.62	0.62	-1859.32			H0	0.38	0.38	-1067.31		
H1	0.65	0.21	-1858.65	1.34	0.25	H1	0.32	**2.14**	-1065.25	4.12	**0.04**
H2	0.64	0.15	-1858.59	1.46	0.23	H2	0.29	0.69	-1065.96	2.70	0.10
											
**Test for *A. melas + A. merus***						**AGAP005196**^**§ **^**- branch test (foreground ω> 1)**					
						
H0	0.62	0.62	-1859.32			H0 (ω = 1)	0.32	1.00	-1065.54		
H1	0.61	0.72	-1859.28	0.08	0.78	H1	0.32	**2.14**	-1065.25	0.59	0.44
H2	0.60	0.71	-1859.24	0.15	0.69						

**AGAP005195 - branch-site test for *A. melas *+ *A. merus***						**AGAP005196 - branch-site test for *A. melas***					

Model A1 (ω = 1)	-	-	-1809.83			Model A1 (ω = 1)	-	-	-1053.14		
Model A (ω variable)	-	-	-1803.44	12.78	**3.51 × 10**^-4^*	Model A (ω variable)	-	-	-1057.53	8.77	0.00*
Sites with *d*_*N*_*/d*_*S *_> 1	71					Sites with *d*_*N*_*/d*_*S *_> 1	-				

ω > 1 and *p *values < 0.05 are in bold. Only sites identified by BEB as statistically significant for *d*_*N*_*/d*_*S *_>1 are reported

* significant after Bonferroni's correction [*p *< 0.016 (0.05/3)]; ^§ ^computed on a reduced dataset (without *A. arabiensis *and *A. quadriannulatus *samples)

The results of the branch-site tests were negative in all cases, except when the branch leading to *A. melas *was assigned as foreground on the gene tree obtained after excluding *A. arabiensis *and *A. quadriannulatus *(*p *< 0.016 after Bonferroni's correction), although BEB failed to identify positive selective sites.

### 3D models

Our analysis indicates that AGAP005194, AGAP005195 and AGAP005196 are chymotrypsin-like serine proteases, a class of enzymes characterized by the presence of a catalytic triad (Serine, Histidine and Aspartate amino acids) and composed by two juxtaposed barrel domains, with the catalytic residues bridging the barrels [23-27]. Residues from N to C terminus of the protease polypeptide substrate are usually named Pi, ..., P3, P2, P1, P1', P2', P3', ..., Pj. The cleavable bond is located between P1 and P1', P1 being the strongest specificity determinant in the majority of the cases. The specificity pocket and the oxyanion hole represent two essential structural features of serine proteases. The former recognizes the side chain of the P1 residue, whereas the latter stabilizes the negative charge that develops on the carboxyl oxygen of the substrate.

**Figure 4:**
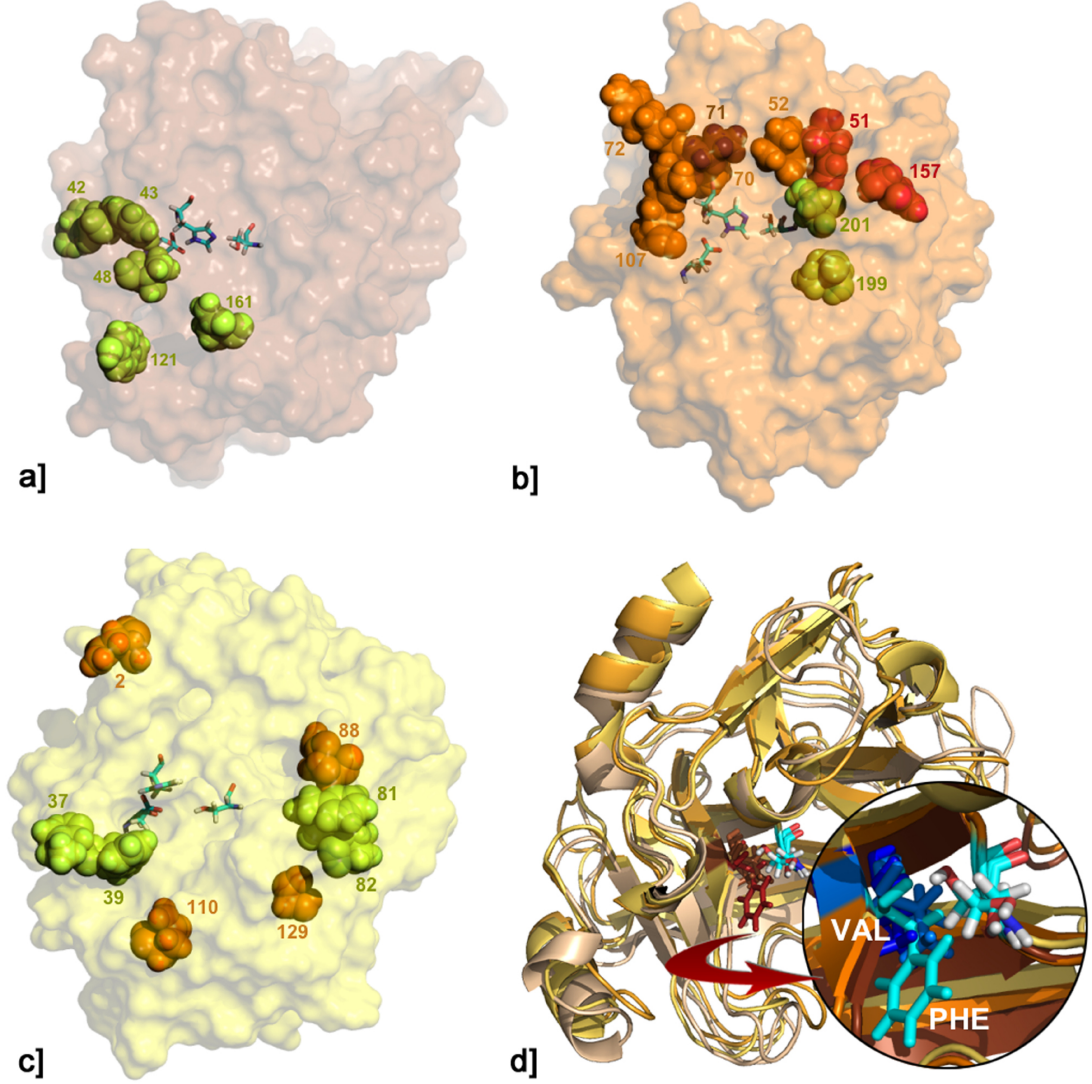
**Residues subjected to positive selection and species or group-specific replacements on 3D models of *Anopheles gambiae *female serine proteases**. Only residues that are within 15 Å and/or have a position that is compatible with a role in the substrate recognition and/or binding, are represented. The catalytic triad is coloured by element. a) AGAP005194: 5 out of the 8 residues subjected to positive selection (Table 2) are reported (green). The single *A. merus *specific residue is > 20 Å distant from the catalytic serine, exposed to the solvent and lies on the face diametrically opposed to that of the catalytic triad (not shown); b) AGAP005195: positive selected residues are in green; group-specific (*A. merus *+ *A. melas*) residues are in orange; residues that are both group-specific and subjected to positive selection are in red. Codon 71 is shown in brown; c) AGAP005196: positive selected residues are in green; group-specific (*A. melas-like*) residues are in orange; d) Superimposition of the 3 proteases models: AGAP005194 in brown, AGAP005195 in orange, AGAP005196 in yellow, residues at position 156 in red (the catalytic serines of the three proteases are reported for reference only). A zoom of the protease specificity pocket (circled), which is occupied by the aromatic ring of the AGAP005194 phenylalanine (phe) in position 156 (in cyan). The less bulky valines (VAL) of AGAP005195 and AGAP005196 are reported in light and dark blue, respectively.

We analysed the specificity pocket of the three enzymes (Figure 4 and Additional file 4). It can be noticed that two particular positions in the specificity pocket are strictly conserved in all proteases, namely a glycine in position 189 (chymotrypsin numbering scheme) and an aspartate in position 226. Even if the other positions are more variable, a noticeably large hydrophobic residue is present in position 213 of all proteases. This suggests that these enzymes may recognize a hydrophobic amino acid in P1, possibly a phenylalanine, which could fit into the pocket (data not shown). Interestingly, AGAP005194 displays a bulky aromatic residue (Phe) in position 213, whereas in the same position both AGAP005195 and AGAP005196 have a smaller hydrophobic one (Val) (Figure 4d and Additional file 4). This difference might in principle correspond to a different substrate preference of AGAP005194 with respect to the other two proteases.

We next determined the position on the protease surface of the residues subjected to positive selection (Figure 4a-c). Interestingly, most of them lie on the surface area of the protein containing the active site and, even if their geometric distribution is rather different in the three proteases, they surround the active site in two out of three cases. By analyzing the distance distribution of the positive-selected residues from the catalytic serine and their position relatively to the catalytic triad, we observed that 5 out of 7 residues in AGAP005194 and 4 out of 8 residues in AGAP005195 are within a close distance (< 15 Å) (Figure 4a,b). In particular, five sites identified as evolving under selective pressures (Table 2 and 3) are in strategic locations: in fact, sites 43 and 48 in AGAP005194 and sites 71, 199 and 201 in AGAP005195, lie on the edge of the specificity pocket (Figure 4a,b). These sites could have an important role in substrate recognition and/or binding. It is worth to note that codon 71 of AGAP005195 has been identified by the branch-site model as evolving under selective pressures along the branch separating the *A. melas *and *A. merus *clade from the other members of the *A. gambiae *complex. Finally, most of the 13 positively selected residues identified for AGAP005196 appears to be "randomly" distributed in the protein structure (data not shown). 4 of these residues (37, 39, 81, 82) are close to each other and lie on the same protein face of the catalytic triad (Figure 4c) and among them 39, 81 and 82 are located near the edge of the specificity pocket. However, the putative high level of ancestral replacement polymorphisms could have led to an overestimation of ω by site-models for most of AGAP005196 positive selected sites (see also Figure 3).

### Immunofluorescence and confocal analysis

**Figure 5:**
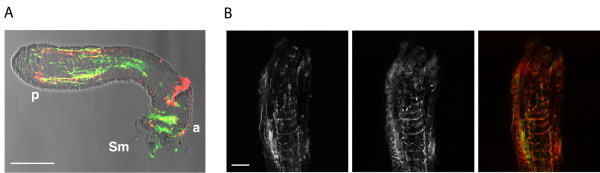
**Immunofluorescence (IF) confocal analysis of *Anopheles gambiae *mating plugs dissected from freshly mated females**. IF reveals that AGAP005194 is found on the surface of mating plug: a) a mating plug showing staining with AGAP005194 (green) and Plugin (red), the major mating plug protein. The two proteins show a good level of co-localization. Letters 'a' and 'p' stand for anterior and posterior, the first and last part of the mating plug to enter the female atrium, respectively; 'Sm' stands for sperm mass, comprising sperm that did not enter the spermatheca and remained attached to the anterior part of the plug. Scale bar: 80 μm. b) A magnification of the posterior tip of the plug, showing staining with AGAP005194 (left), Plugin (centre) and the overlay (right). Scale bar: 20 μm

To gain further support for the hypothesis that the atrium-expressed proteases are involved in the digestion of the mating plug, we used polyclonal antibodies against AGAP005194 in immunofluorescence (IF) experiments on mating plugs dissected from recently mated *A. gambiae *females. The IF experiments verified that indeed this female protease is found on the surface of the plug after mating (Figure 5a). Moreover, the pattern of AGAP005194 on the plug surface closely matched the pattern of Plugin, the major plug protein (Figure 5a,b) [8]. We also confirmed that by 24 hours post-mating just some traces of AGAP005194 protein are left in the female atrium compared to virgin levels (data not shown). All together, these data strongly suggest that AGAP005194 (and probably the other atrial-specific protease AGAP005195) is completely dedicated to proteolytic activities related to copulation.

## Discussion

Here we present data on a cluster of 3 female LRT-specific serine protease genes suggested to be involved in post-mating processes in *A. gambiae *s.s. As already shown for other genes with different functions, the reconstruction of the 3 gene-trees shows that most species share alleles at all loci, as an effect of introgression and/or retention of ancestral polymorphisms, and that only *A. merus *and *A. melas *are placed in monophyletic assemblages (Figure 2). On the other hand, we found an unusually high substitution rate, which contributes mostly to an exceptionally high level of intra-specific polymorphisms, especially at nonsynonymous sites (Table 1). Moreover, while *A. gambiae*, *A. arabiensis*, and *A. quadriannulatus *do not differ for any fixed replacement, *A. melas *and *A. merus *diverge from the other species at all loci, showing a high number of fixed substitutions at both synonymous (7-9) and nonsynonymous (13-23) sites at locus AGAP005195.

The comparisons of different site-models - used to test for the presence of positively selected sites in our codon alignments - indicate that in all 3 serine proteases most amino acidic residues (75-87%) are conserved among the species analysed and that these proteins are overall subjected to purifying selection (SLAC global *d*_*N*_*/d*_*S *_= 0.482, 0.672, 0.406 for AGAP005194, AGAP005195, AGAP005196 respectively, see also figure 3). However, a number of codons appear to be targeted by positive selective pressures in all genes (Tables 2 and 3) and, noteworthy, some of them lie relatively close to the catalytic triad and/or on the edge of the specificity pocket, which is considered to be important for substrate recognition and/or binding (Figure 4a-c).

Moreover, lineage-specific tests of adaptive evolution detected events of episodic selection in AGAP005195 and AGAP005196. In the former gene, the branch-site models detected episodic selection on lineages that were not detected by branch-models, as expected if positive selection occurs at a few sites in an overall purifying selection background. In particular, codon 71 is shown to have evolved under selective pressures along the branch separating the clade grouping *A. melas *and *A. merus *from the other members of the *A. gambiae *complex. These two species are grouped apart also on the basis of other substitutions at positions 51, 52, 70, 72, 107, 157 and 193. Among them, sites 51 and 157 were also detected by BEB analysis of site-model comparisons (Table 1). It is worth to note that most of these residues are exposed to the solvent and form a sort of semi-circle around the active site (Figure 4b), suggesting that this epitope might interact with a peculiar substrate in *A. melas *and *A. merus*. It can be hypotesized that this epitope evolved under selective pressures in a common ancestor of these two species, or, alternatively, because of convergent evolution. The latter hypothesis would be consistent with the distant phylogenetic relationship between these two species, as suggested by their chromosomal inversions patterns [11,28]. In addition, we found *A. merus*- (13, 36, 104, 105, 152, 155, 159, 160, 165, 183, 199) and *A. melas*- (152, 181) specific replacements: among them, residues in position 152, 155, 159 and 199 (the last identified by all ML selection methods) on the same protein side of the catalytic triad, while the remaining residues are located in exposed loops in regions far from it (not shown in Figure 4). Follow up molecular studies will be needed to clarify whether AGAP005195 has evolved a different role in *A. melas *and *A. merus *as opposed to the other species of the complex.

In AGAP005196, the branch model detected episodic selection along the branch separating the *A. melas*-like from the *A. gambiae*-like alleles, and along the branch leading to *A. melas*. In both cases, the ω assigned to these branches (assumed to be the same at all sites) was not significantly >1. On the contrary, the branch-site test applied to the branch leading to *A. melas *was statistically significant, but BEB failed to identify sites under positive selection. Finally, a significant excess of fixed replacements between *A. gambiae *and *A. melas *was also detected by the MK-test for this gene (Table 1).

The interpretation of the results obtained is not straightforward, with particular reference to those from the models of episodic selection. As already mentioned, the occurrence in AGAP005196 of relatively high frequencies of nonsynonymous mutations at both intra- and inter-specific levels may affect the interpretation of evidence of episodic selection along the branch leading to *A. melas*. In addition, although *A. melas *presents four species-specific amino acid replacements at positions 2, 88, 110, 129 (which also contribute to the significance of MK test in the comparison with *A. gambiae*), these are shared with some *A. arabiensis *and *A. quadriannulatus *individuals, thus revealing again a pattern of incomplete lineage sorting also for this particular haplotype.

Increased *d*_*N*_*/d*_*S *_among conspecifics (or among closely related sequences/species) has been observed in other taxa, and a variety of hypotheses has been suggested to interpret these results under a regime of negative selection: balancing selection, variable population sizes, variable mutation rates, relaxed selective constraints and/or the prevalence of slightly deleterious mutations [19,20]. Based on our data, we cannot rule out any of these hypotheses, nor provide an unambiguous explanation for the observed pattern. However, the high level of replacement polymorphisms observed in all the 3 serine proteases (Table 1) suggests that these genes might evolve as a functionally redundant cluster. In fact, duplicated genes with partially or completely overlapping functions may experience relaxed evolutionary constraints that allow them to rapidly explore and eventually fix new advantageous variants [29-31]. Indeed, in some *Drosophila *species, female-expressed serine proteases have experienced recurrent events of lineage-specific gene duplications immediately followed by a period of positive selection, indicating neo-functionalization of gene duplicates [32,33]. Evidence of recent duplication activity playing a crucial role also in the evolution of *Anopheles *female proteases is provided by the finding of an additional copy of AGAP005196 located in the same gene cluster. This previously undetected paralog bears the insertion of a miniature transposable element of the TA-I-α-Ag MITE family, which is frequently associated with gene introns and putatively affects gene regulation [17,34]. If we assume that the *A. gambiae *serine-protease cluster is experiencing relaxed evolutionary constraints, the decrease of *d*_*N*_*/d*_*S *_observed only at increasing evolutionary distances in all 3 proteases (Figure 3) may simply reflect a lag in removal of slightly-deleterious mutations by purifying selection occurring in the early stages of sequence differentiation. This could, for instance, explain the ω >>1 observed for most *A. merus *intra-specific comparisons in AGAP005194 (Figure 3), which could alternatively be interpreted also as the result of long-term positive selective pressures in this species (Table 3). The latter interpretation is consistent with the observation that some *A. merus*-specific polymorphic replacements (i.e. 42 and 43, identified by M2 and M8) map close to the catalytic triad (Figure 4a), although some of them are also shared with other species (e.g. *A. quadriannulatus*). Hence, a better knowledge on the functional importance of these non-synonymous changes would be needed to evaluate if balancing selection is maintaining different haplogroups at intermediate frequencies in the *A. merus *gene pool.

In addition, we cannot exclude that genetic drift caused by long-term small effective population sizes of *A. melas *and *A. merus *might have also contributed to determine the observed fixation of species-specific substitutions (and, therefore, lineage-sorting). Indeed, it has been argued by many authors that coalescence processes and demographic fluctuations have differently affected and shaped the population genetic history of members of the *A. gambiae *complex [35-38]. Since π_s _can be used as an estimator of 4Neμ, assuming the same mutation rate (μ) in all lineages, differences at neutral sites at the AGAP005195 locus would indicate that *A. gambiae *and *A. arabiensis *have experienced larger effective population sizes than *A. merus*, consistently both with their wider geographic range and their higher levels of shared ancestral polymorphisms (see above: πs ~ 1.7%, 0.4%, and 0.0% for *A. gambiae*, *A. arabiensis *and *A. merus*, respectively). A similar explanation could be applied to the four species-specific replacements found in *A. melas *at the AGAP005196 locus and indeed, under the same assumption, π_s _would indicate that *A. gambiae *and *A. arabiensis *have experienced larger effective population sizes than *A. melas *(π_s _~1.9%, 2.8%, and 0.1% for *A. gambiae*, *A. arabiensis *and *A. melas*, respectively).

Difficulties in the interpretation of results from selection-tests are mostly due to the unresolved phylogenetic history of the *A. gambiae *complex, as already argued for genes modulating immune responses to the malaria parasites [15,35,39-43]. However, the accurate inferences of the structural models of these proteins allowed us to better evaluate the importance of these putative positive selected residues. It is worth to note that most positive selected residues appear not located "at random" on 3D structures. In particular, the structural models show that AGAP005194 and AGAP005195 positive selected residues are placed in a relatively large surface area near the catalytic triad and in the specificity pocket (Figure 4). A similar pattern was also observed for duplicated genes encoding several mating-induced female serine proteases of *Drosophila *[32,33,44-47], which are supposed to interact with rapidly evolving accessory gland proteins transferred by males during mating [48-50]. Sexual selection and/or conflict due to male-female protein interactions have been considered to be responsible for these patterns in *Drosophila *and to have promoted rapid divergence, which in some cases has been found to be ten-fold higher than in genes expressed in non-reproductive tissues [46]. However, in the case of the monandrous species of the *A. gambiae *complex, sexual selection and/or conflict cannot be convincingly invoked to explain the presence of positive selected sites near the catalytic triad or in the specificity pocket. A more likely explanation could be that the observed pattern is derived from the interaction of the 3 female-specific proteases with rapidly evolving substrates or inhibitors that may differently modulate their catalytic activity. This scenario mirrors the rapid evolution of immune-related genes engaged in host-pathogen arms race (see ref. 35 and reference therein). Indeed, in *Drosophila *females sexually antagonistic interactions at the time of mating activate a number of immune-related genes, which are induced by the transfer of sperm and seminal fluid peptides, rather than by pathogens [51]. It has been suggested that this immune response could account for the 'cost of mating', in the form of decreased female lifespan and fecundity [51]. Although in *A. gambiae *no significant induction of known immune genes was detected after mating and no cost of mating has ever been reported, some genes encoding immune-like peptides were shown to be strongly upregulated in the female atrium [14]. It could be hypothesized that the three serine proteases studied here have a dual role in *Anopheles *fertility: helping the preservation of the female reproductive tract from possible damaging factors transferred during mating, and processing of the mating plug. In effect, a dual role could be hypothesized for AGAP005194: this protease has been found to respond to bacterial infection [52] and its role in processing the mating plug is confirmed by its localization on the plug surface (Figure 5). Given the strong down-regulation of the 3 serine proteases at 24 h post mating, it is reasonable to speculate that their transcription may be turned down by male-derived factors released during mating plug digestion, thereby reducing the cost of mating and allowing females to entirely divert their energy resources to reproductive processes. This hypothesis would be more consistent with a co-operation between the sexes in optimizing their reproductive success, rather than with an arms race among sexes.

The relaxation in purifying selection provided by the functional redundancy in the cluster would allow the maintenance of high genetic variability, on which positive selection could act to eventually fix novel variants to perform either new or more specific functions (neo-functionalization) [29]. In fact, the 3D models highlighted an important structural differentiation in the two atrium-specific proteases AGAP005194 and AGAP005195 that might have a different substrate preference (Figure 4d and Additional file 4). This differentiation, due to a bulky aromatic residue (Phe) in position 213 of AGAP005194 respect to a smaller hydrophobic one (Val) in AGAP005195 and AGAP005196, is fixed in all species of the *A. gambiae *complex and thus likely appeared very early during the evolution of the paralogs, probably because of neo-functionalization. The relative high differentiation (35-50% identity on PEST genome ver. 3.5, Sept. 2009) and the absence of gene conversion among the three paralogs (except for the 'recent' copy of AGAP005196 bearing the MITE insertion) indicate that these are not at their very early stages of duplication in the *A. gambiae *complex. In this context, it would be important to obtain more information on the conservation of gene linkage (synteny) for this cluster of functionally related genes in species more distant to those within the *A. gambiae *complex. Interestingly, a comparative study of gene orders between *A. gambiae *and *A. stephensi *at ~1 Mb resolution did not detect a conserved synteny block for the chromosome region containing these serine protease genes [53]. Furthermore, the order of these genes was inverted (if not reshuffled) in *A. stephensi *because of the accumulation of a large number of fixed inversions during the divergence of the two species. Novel data from the ongoing genome sequencing project from 13 more *Anopheles *species will provide a better knowledge on the orthology and synteny of these genes and, hopefully, a stable phylogenetic framework to trace the evolution of relevant amino acid substitutions in copies of this gene cluster. These data would also help validating the findings of other 'novelties' such as fixed autapomorphic and synapomorphic replacements located in strategic positions close to the catalytic triad of the 3 proteases, which suggests an interaction with factors transferred by males (e.g. substrates, inhibitors or pathogens) that may have differently evolved in independent *A. gambiae *lineages. In addition, the spermatheca-specific protease AGAP005196, because of its relative low divergence among members of the *A. gambiae *complex, is likely to have appeared more recently than the other copies with functions partially or completely overlapping with those of AGAP005195. Indeed, preliminary data show that a number of seminal proteins are transferred to the spermatheca bound on sperm (Catteruccia F., personal communication), as it has been shown in *Drosophila *[54]. It is possible that AGAP005196 is also experiencing relaxed evolutionary constraints that allow the accumulation of mutations not tolerated in the previous selective regime and that might be responsible for its adaptation to substrates present in a novel reproductive tissue (i.e. the spermatheca). This is consistent with the observation that *A. melas*-specific sites 88 and 129 are located nearby the edge of the specificity pocket and might play a role in substrate recognition and/or binding (Figure 4c).

## Conclusions

To summarize our results, we found i) an unusually high level of replacement polymorphisms, ii) evidence of a recent gene duplication activity, iii) species/group-specific fixed replacements, iv) sites evolving under long-term and episodic positive selection, v) structural differences among proteases putatively affecting their substrate specificity and, vi) additional evidence of a direct role of these proteases in mating plug digestion.

Overall, our data unveil an unexpectedly intricate evolutionary scenario for these 3 *Anopheles *female-expressed serine proteases. Unfortunately, as already remarked, the intricated phylogenetic history of the *A. gambiae *complex hinder the interpretation of our results from selection-inference methods. Nevertheless, the 3D structure study of these proteins allowed us to highlight a closeness of most of positive selected sites to the catalytic triad and/or to the edge of the specificity pocket. Thus, despite the possible presence of false positives in site-based tests of selection, the identification of replacements in amino acid positions that are crucial for the activity of these proteases (especially if maintained by long-term balancing selection at least in some species) encourages further investigation on the role of these residues in substrate recognition or binding in *Anopheles *female serine proteases.

Further experimental analyses in the other species of the *A. gambiae *complex will be also needed to assess whether the patterns of evolution observed for these proteins might correlate to diverse biological functions. If a relevant role of the 3 serine proteases in the reproductive success of *A. gambiae *species will be highlighted, this would open perspectives for the development of innovative strategies aimed at limiting the fertility of these mosquitoes, and ultimately contribute to control malaria transmission.

## Methods

### Field collected samples

Evolutionary analyses were carried out on 5 species of the *A. gambiae *complex. Samples of both incipient species of *A. gambiae *s.s. - namely the M- and S- molecular forms - were considered in our study. Sampling on a wide geographic scale was planned to increase the power to distinguish between polymorphisms and fixed differences among species. Specimens were collected in several localities along the geographical distribution of each species (Additional file 5): *A. gambiae *s.s. M- and S-form adults were collected between 1998 and 2008 in 6 African countries (Angola, Benin, Cameroon, Ivory Coast, Tanzania, Zimbabwe), *A. arabiensis *from 5 countries (Senegal, The Gambia, Angola, Zimbabwe and Kenya), *A. melas *from Angola, Gabon and Guinea Bissau, *A. quadriannulatus *A from Zimbabwe and Malawi and *A. merus *from Mozambique and Tanzania. Sequences of AGAP005194, AGAP005195 and AGAP005196 genes were obtained from a total of 51, 48 and 61 individuals, respectively (8, 7 and 7 for *A. gambiae *M-form; 9, 11 and 11 for *A. gambiae *S-form; 10, 8 and 15 for *A. arabiensis*; 7, 7 and 8 for *A. quadriannulatus*; 8, 9 and 12 for *A. melas*; 7, 6 and 8 for *A. merus*). Species names are abbreviated as follows: *A. arabiensis *= AR; *A. gambiae *M form = GA-M; *A. gambiae *S form = GA-S; *A. melas *= ML; *A. merus *= MR; *A. quadriannulatus *A = QD.

### DNA methods

Genomic DNA was extracted using standard procedures and specimens were identified to species/forms using both PCR-RFLP and *SINE200 *methods [55,56]. Primers were designed using Primer3 program [57] in order to amplify part (~700-900 bp) of AGAP005194, AGAP005195 and AGAP005196 loci (Figure 1). To successfully amplify the targeted portions, a nested PCR protocol was applied in most cases: the PCR products obtained in the first round were diluted 1:100 and used as templates for subsequent PCR reactions with internal primers (Additional file 6). PCR were performed in a 25 μl reaction which contained 1 pmol of each primer, 0.2 mM of each dNTP, 1x reaction buffer, 1.5 mM MgCl_2_, 2.5 U Taq polymerase (Bioline), and 0.5-1.0 μl of template DNA extracted from head and torax of a single mosquito. Thermocycler conditions were: 94°C for 10 min followed by 35 cycles of 94°C for 30 sec., 50-54°C for 30 sec. and 72°C for 1 min., with final elongation at 72°C for 10 min. The resulting products were analysed on 1% agarose gels stained with ethidium bromide, purified using the SureClean Kit (Bioline) and sequenced at the BMR Genomics s.r.l. (Padua, Italy). Sequences were deposited in GenBank under Accession Numbers HQ332601-HQ332768.

### Sequence editing and codon alignments

All sequences were edited using the Staden Package ver. 2003.1.6 [58]. Haplotype estimation was performed with the PHASE algorithm [59] implemented in DNAsp v5 [60]. After removing introns, codon alignments were recovered from each protein using MAFFT ver. 5 [61]. To test for gene conversion, we also built multiple codon alignments including alleles sampled from all loci and only merging AGAP005195 and AGAP005196 alignments (see below).

### Polymorphisms, divergence and tree inferences

Basic analyses of genetic polymorphisms and divergence were performed using DnaSP v5 [60]. A bayesian phylogenetic approach was used to infer gene-tree topologies that also served as the basis for the implementation of the maximum likelihood methods of the PAML package ver. 4.3 [62]. The nucleotide substitution models were selected using jModeltest 0.1.1 [63] according to the Akaike Information Criterion for small sample size (AICc) and then used for bayesian inferences on complete and reduced datasets of coding regions using Mr Bayes ver. 3.1.2 [64]. 2.0 × 10^6 ^generations were run and Markov chains were sampled every 1000 generations. To ensure sampling of topologies after chain convergence, the first 1000 trees were discarded as 'burnin' and the remaining trees were used to compute posterior probabilities at nodes. Since introgression and/or retention of ancestral polymorphisms are common in the reconstruction of genealogical relationships among the species of the *A. gambiae *complex [37,65,66], the genealogical sorting index (gsi) [67] was used to quantify the exclusive ancestry of alleles sampled from each species/form of the *A. gambiae *complex on the reconstructed gene trees. Statistical significance of gsi values were assessed using 10000 permutations, as implemented in the web server http://www.genealogicalsorting.org/.

### Tests for adaptive evolution

In order to assess patterns of adaptive evolution on each gene, different approaches were used. The synonymous (*dS*) and nonsynonymous (*dN*) substitution rates were computed using the codon-based model of Goldman and Yang [18] for each pairwise comparison, as implemented in the Yn00 program of PAML 4.3 [62]. The ratio of *dN*/*dS *(= ω) was also calculated for each comparison: under neutrality ω = 1; for genes subjected to functional constraints such that deleterious nonsynonymous amino acid substitutions are purged from the population ω < 1, while for positively selected genes ω >1. This approach can be used to describe the general pattern of selection on a protein, but, since it averages ω over sites and time, it has little power if only a few sites have been targets of adaptive evolution [68].

McDonald-Kreitman (MK) tests [69] were performed for each gene to identify selection on the whole protein through an excess of fixed amino acid substitutions between species. This test compares the number of nonsynonymous and synonymous sites that are polymorphic within a species (P_NS _and P_S_) and fixed between species (F_NS _and F_S_). Under neutrality P_NS_/P_S _= F_NS_/F_S_, whereas positive selection leads to an increase in nonsynonymous fixed divergence (F_NS_/F_S _> P_NS_/P_S_). Statistical significance of MK tests was assessed with DNAsp 5 [60] using the Fisher's exact test.

Maximum likelihood (ML) approaches, which allow ω to vary among codons, were used to perform a site-by-site detection of positive selection. For this purpose, codon alignments and tree topologies were used as input in CodeML of the PAML 4.3 [62]. Two pairs of site models forming two likelihood ratio tests of positive selection (i.e. M1a vs. M2a, and M7 vs. M8) were fitted to our data. In the first comparison, a nearly neutral model (M1a) allowing only two categories of sites with ω = 1 and 0 < ω < 1, respectively, was compared with a selection model (M2a), which allows an additional category of positively selected sites (i.e. ω > 1). In a second test, the M7 model (beta), that allows sites to have different ω estimated from a beta distribution and varying in the interval (0, 1), was compared with an alternative selection model, M8 (beta and ω), which allows to add another category of ω that accounts for positively selected sites (ω > 1). Likelihood ratio tests were used to determine the relative fit of these hierarchically nested models using 2 d.f. (e.g., if M1a/M7 (neutral) can be rejected in favor of M2a/M8 (selection), positive selection is inferred). If tests were significant, the Naïve Empirical Bayes (NEB) and the Bayes Empirical Bayes (BEB) were applied to calculate the posterior probabilities for site classes, and, thus, to identify sites putatively evolving under positive selection.

Branch models of CodeML - that allow ω to vary among branches in a tree - were applied to detect selection acting on a particular lineage. To test for episodic selection ('H_1 _hypothesis'), we designated each branch leading to monophyletic lineages in our trees as foreground branches (i.e. the branch of interest) and branch model 2 (NSsites = 2, model = 0, allowing a free ω for the foreground branch) was compared to branch model 0 (NSsites = 0, model = 0, one ω for all branches, 'H_0 _hypothesis') to test if ω along the foreground branches were significantly larger compared to the ω along all other branches. To test an alternative pattern of long-term change in selective pressures within specific lineages ('H_2 _hypothesis'), we also labeled all branches in a clade as foreground and evaluated the fit of this model to our data.

In order to test if certain codons were under selection in the foreground branches, and thus identify positively selected sites within specific monophyletic lineages in our trees, we also fit the branch-site models. This method can be useful when selective pressures change over time at just a fraction of sites. This test was performed by comparing the modified Model A (model = 2; NSsites = 2) with the corresponding null model, i.e. Model A1 with ω_2 _fixed to 1. In this case, the BEB procedure was used to identify positively selected codons in the foreground branches. Bonferroni's procedure was used to control the family-wise error rate (≤ 0.5%) and correct for multiple testing, as it has been shown to be powerful when the branch-site test is applied without *a priori *hypothesis to multiple branches on a tree [70].

Three additional methods based on ML approach and implemented in HyPhy Datamonkey webserver (URL: http://www.datamonkey.org) [71] were used to compare results with those obtained using CodeML. As in CodeML, these methods, namely the SLAC, FEL and REL methods [71] are based on a site-by-site analysis aimed to identify single aminoacids under positive selection. However, in contrast to CodeML models, these methods estimate *dS *at each codon site, thus taking into account synonymous rate variation among sites. The starting trees that served as the basis for the HyPhy analyses were inferred automatically by the program itself. We chose a significance level of 0.1 for FEL and SLAC methods and a Bayes Factor = 50 for REL analysis.

### Recombination analyses

Site-by-site ML methods implemented in CodeML assume no recombination among sequences. As a consequence, in case of recombination, false evidence of positive selection might arise using these methods [72]. The same effect occurs in case of gene conversion among loci in a gene cluster [73]. Then, we tested for recombination (and gene conversion) in our datasets by: i) a scan for recombination using the 7 methods implemented in the RDP3 software [74] using default settings (i.e. RDP, Bootscanning, GENECONV, MaxChi, Chimaera, SiScan, 3SEQ), ii) the Genetic Algorithm Recombination Detection (GARD) method implemented in the Web interface of HyPhy Datamonkey [75].

### Generation of 3D protein models

The 3D models of the three proteases were built using comparative modelling techniques and employing the complete sequence derived from the *A. gambiae *genome. In order to build reliable homology models and analyze the structural context of residues under selective pressures, we used the HHpred web-server (http://toolkit.tuebingen.mpg.de/hhpred) [76] to identify suitable templates and obtain their sequence alignment with the target protease protein sequences. This tool is based on the comparison of a Hidden Markov Model (HMM) describing the family of the target proteins with HMMs built for each protein of known structure. For each target, the ProMals3D tool [77] was used to optimize the target-template alignment which was then used as input for the Modeller package [78]; Modeller uses distance constraints derived from the template(s) to build consistent models of the target protein. We decided to generate 50 models for each target protein.

For AGAP005194 HHpred identified three proteins of known structure as best templates: fire ant chymotrypsin, PDB code: 1eq9, sequence identity 38%, E-value less than 1.4E^-45 ^[79], fiddler crab collagenase, PDB code: 1azz, sequence identity 31%, E-value < 1.4E^-45^) [80] and crayfish trypsin, PDB code: 2f91, sequence identity 32%, E-value < 1.4E-^-45^) [81]. The best templates for AGAP005195 were fire ant chymotrypsin (sequence identity 36%, E-value < 1.4E^-45^) [79] and fiddler crab collagenase (sequence identity 28%, E-value < 1.4E^-45^) [80]. One template was identified for AGAP005196, i.e. fire ant chymotrypsin, PDB code: 1eq9 (sequence identity 28%, E-value < 1.4E^-46^) [79]. The 50 models obtained for each target protein were ranked first according to the Modeller Objective Function and then to the Modeller DOPE scoring function [82]. The best 10 models in each ranked set were evaluated using the ProQ server [83] and, finally, the ProQ best five models re-evaluated using the MQAP MetaServer [84]. The MQAP best scoring model was selected as the final one. Three-dimensional analysis and visualization were carried out using the VMD (http://www.ks.uiuc.edu/Research/vmd/) and PyMol software tools (http://www.pymol.org/). Notice that the target-template sequence identity ranged between 28% (AGAP005196-1eq9) and 38% (AGAP005194-1eq9) and that the most effective methods for protein structure prediction (HHPred followed by model building with Modeller) was used. In recent blind tests in the context of the Critical Assessment of Techniques for Protein Structure Prediction (CASP), this strategy revealed to be extremely effective (http://predictioncenter.org). In our specific case, it guarantees that the expected difference between the models and the real structure of the proteins is of the order of 0.5 - 1.0 Å root mean square deviation on the main chain atoms of the conserved regions. This implies that the unavoidable deviations of the models from the native structures would not significantly affect the position of the specificity pocket residues and, therefore, would not alter the conclusions of our structural analysis.

### Immunostaining and confocal analysis of mating plug

Mating plugs from recently mated *A. gambiae *females were dissected on ice and fixed in PBS 4% formaldehyde solution. After washing in PBS, the samples were incubated with 2% hydrogen peroxide to reduce autofluorescence, washed in PBS and then blocked and permeabilized in PBS with 1% BSA and 0.03% Triton X-100. Then the samples were incubated with 1.5 μg/ml anti-AGAP005194 in blocking buffer, washed, incubated with 2 μg/ml anti-Plugin [8], washed and finally stained with anti-mouse Alexa 488 and anti-rabbit Cy3 (Invitrogen) at a 1:1,000 dilution. Tissues were then mounted in DAPI-containing Vectashield medium (Vector Laboratories, Inc.) and visualized using a Leica SP5 inverted confocal microscope. Affinity-purified polyclonal antibody against AGAP005194 was raised in mouse against a peptide epitope (CGTSPAKLQTINAPS) by a commercial supplier (GenScript Corp., Piscataway, NJ).

### Fluorescence *in situ *hybridization (FISH)

To understand whether AGAP005196 and the novel identified paralog of AGAP005196 with the MITE insertion are clustered together (see Results), we designed a 557 bp probe [using primers RG5196-FISH-f (5'ACGGGTGGGAACAAATGATA3') and RG5196-FISH-r1 (5'CCAACTGACTACGCCAACCT3')] that included partial sequences of AGAP005196 exons 2 and 4, as well as full sequences of exon 3 and introns 2 and 3. Because the MITE was found within intron 3 of the paralog gene, this probe binds predominantly to the common sequences (intron 2 and exon 3) of both AGAP005196 and its paralog. In order to ensure that the presence of the MITE did not interfere with the overall efficiency of the binding, we designed an additional 426 bp probe [using as alternative reverse primer RG5196-FISH-r2 (5'ACCATGCCCTGCTCTAGAAA3')] that included partial sequences of exons 2 and 3, as well as a full sequence of intron 2, but not the third intron. The genomic DNA of single *A. gambiae *SUA mosquitoes was extracted with the Wizard SV Genomic Purification System (Promega Corporation, Madison, WI, USA) and used as a template for PCR. PCR products were gel purified using the Geneclean kit (Qbiogene, Inc., Irvine, CA). Chromosomal preparations were made from the ovaries of half-gravid females of the SUA strain of *A. gambiae*, the OPHANSI strain of *A. merus*, and the DONGOLA strain of *A. arabiensis*. The *in situ *hybridization procedure was conducted as previously described [85]. The DNA was labeled with Cy3-AP3-dUTP (GE Healthcare UK Ltd., Buckinghamshire, England) using Random Primers DNA Labeling System (Invitrogen Corporation, Carlsbad, CA, USA). DNA probes were hybridized to the chromosomes at 39°C overnight in hybridization solution (Invitrogen Corporation, Carlsbad, CA, USA). Then the chromosomes were washed in 0.2 × SSC (Saline-Sodium Citrate: 0.03 M Sodium Chloride, 0.003 M Sodium Citrate), counterstained with YOYO-1, and mounted in DABCO. Fluorescent signals were detected and recorded using a Zeiss LSM 510 Laser Scanning Microscope (Carl Zeiss MicroImaging, Inc., Thornwood, NY, USA).

## Authors' contributions

Conceived and designed the experiments: EM, AT, FC, AdT. Performed the experiments and analyse the results of: i) genetic data: FT, EM, ii) fluorescence *in situ *hybridization: PG, IVS, iii) immunofluorescence and confocal analysis: FB, FC, iv) 3D protein structure modelling: AV, DR Wrote the paper: EM, FT, AV, IVS, PA, AT, FC, AdT. All authors contributed to and approved the final manuscript.

## Supplementary Material

Additional file 1 **Features of the novel identified paralog of AGAP005196 bearing the MITE insertion**. Nucleotide alignment of the identified novel paralog of AGAP005196 containing a 368 bp insertion of a miniature inverted repeat transposable element (MITE) of TA-Iα-Ag inside the third intron [TIR = terminal inverted repeats; W = A/T in *A. arabiensis *15.2] and putative secondary structure of the inserted MITE, base-pairs probability (from blue = 0 to red = 1) and minimum-free energy. Click here for file

Additional file 2 **FISH of AGAP005196 to polytene chromosomes of *A. gambiae*, *A. arabiensis*, and *A. merus***. Top panel: FISH of the AGAP005196 probe encompassing the 3rd intron (left) and the AGAP005196 probe excluding the 3rd intron (right) to polytene chromosomes of *A. gambiae*. Bottom panel: FISH of the AGAP005196 probe encompassing the 3rd intron to polytene chromosomes of *A. arabiensis *and *A. merus*. Arrows indicate the single site of hybridization in the division 21E of the 2L arm. Click here for file

Additional file 3 **Genetic polymorphisms**. Nucleotide polymorphisms of AGAP005194 (= 489 bp), AGAP005195 (= 603 bp), AGAP005196 (= 456 bp) computed using DNAsp ver. 4. Click here for file

Additional file 4 **Scheme of the specificity pocket of the three serine proteases**. Residues that contribute to the shape of the pocket are represented with filled circles. Residue type and positions in the AGAP005194, AGAP005195 and AGAP005196 proteases are separated by a "/". Red numbers in brackets indicate the residue position according to the chymotrypsin numbering scheme. Arrows indicate the predicted direction of the residue side chain as deduced by reconstructed models, with the length of the arrow being proportional to the size of the side chain. Click here for file

Additional file 5 **Map of sampling localities**. Numbers in parenteses and species abbreviations after collection sites were used to indicate species and geographic origins of sequence vouchers submitted to GenBank. Sequences from 1 to 8 individuals per species for all genes were obtained from each locality [except for GA-M from Benin (AGAP005194 only), GA-S from Tanzania and AR from The Gambia (AGAP005194 and AGAP005195 only)]. Click here for file

Additional file 6 **Primer table**. Sequences of primers used for the amplification of selected portions of female serine protease genes. Click here for file
