# Supplementary figures and images for: Molecular evolution of a gene cluster of serine proteases expressed in the Anopheles gambiae female reproductive tract
Source: BMC Evol Biol. 2011 Mar 19;11:72. doi: 10.1186/1471-2148-11-72 (PMC3068966; doi:10.1186/1471-2148-11-72)

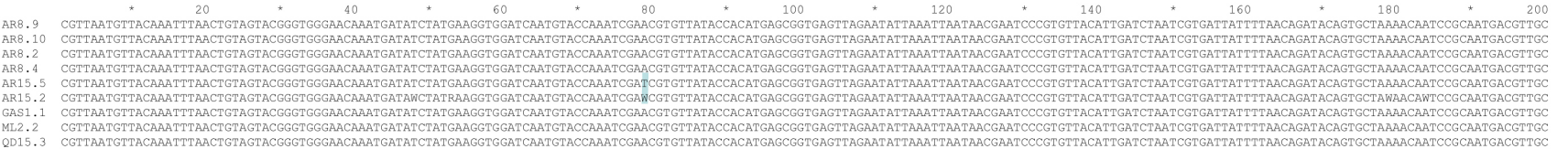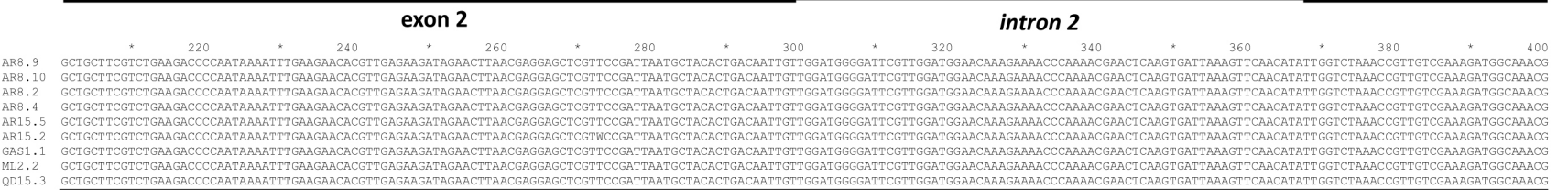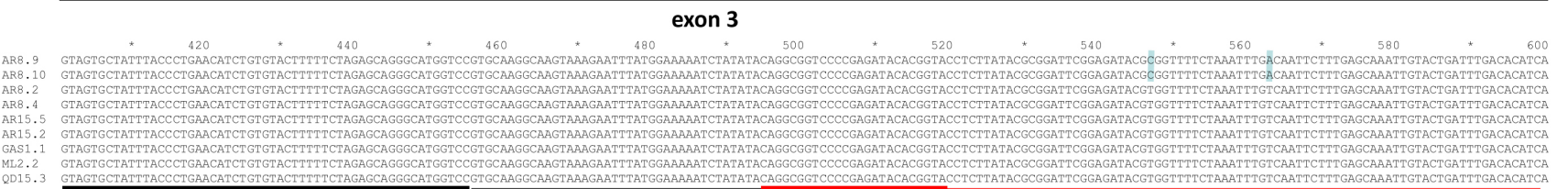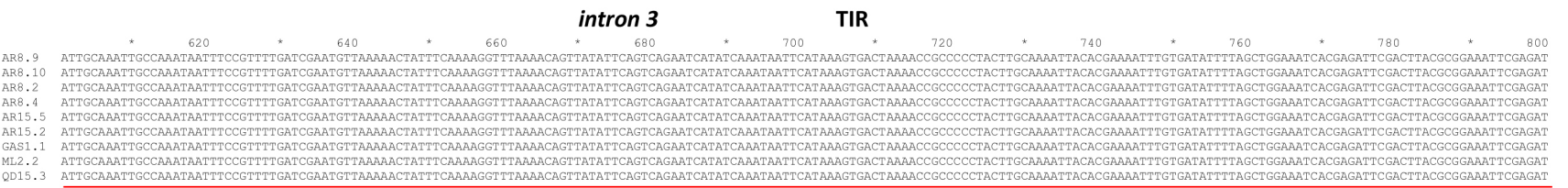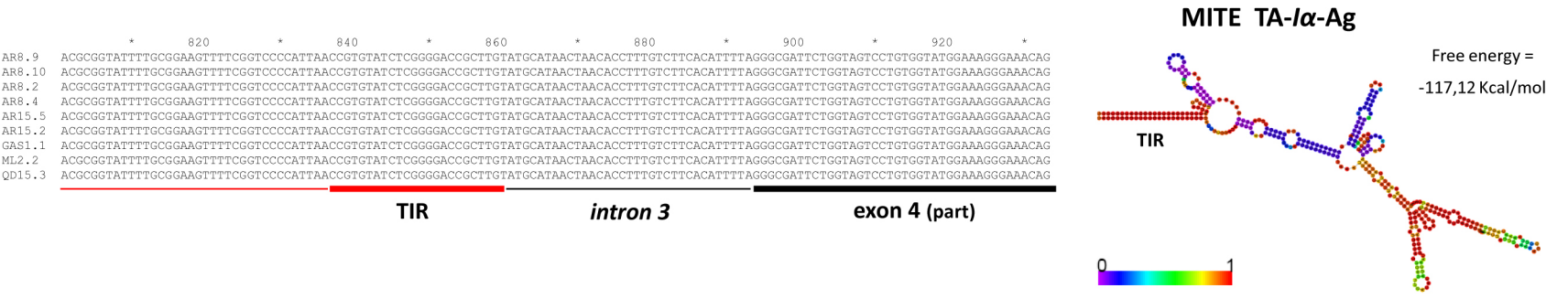

Supplement: Additional file 1 — Features of the novel identified paralog of AGAP005196 bearing the MITE insertion. Nucleotide alignment of the identified novel paralog of AGAP005196 containing a 368 bp insertion of a miniature inverted repeat transposable element (MITE) of TA-Iα-Ag inside the third intron [TIR = terminal inverted repeats; W = A/T in A. arabiensis 15.2] and putative secondary structure of the inserted MITE, base-pairs probability (from blue = 0 to red = 1) and minimum-free energy. [file 1471-2148-11-72-S1.PDF]

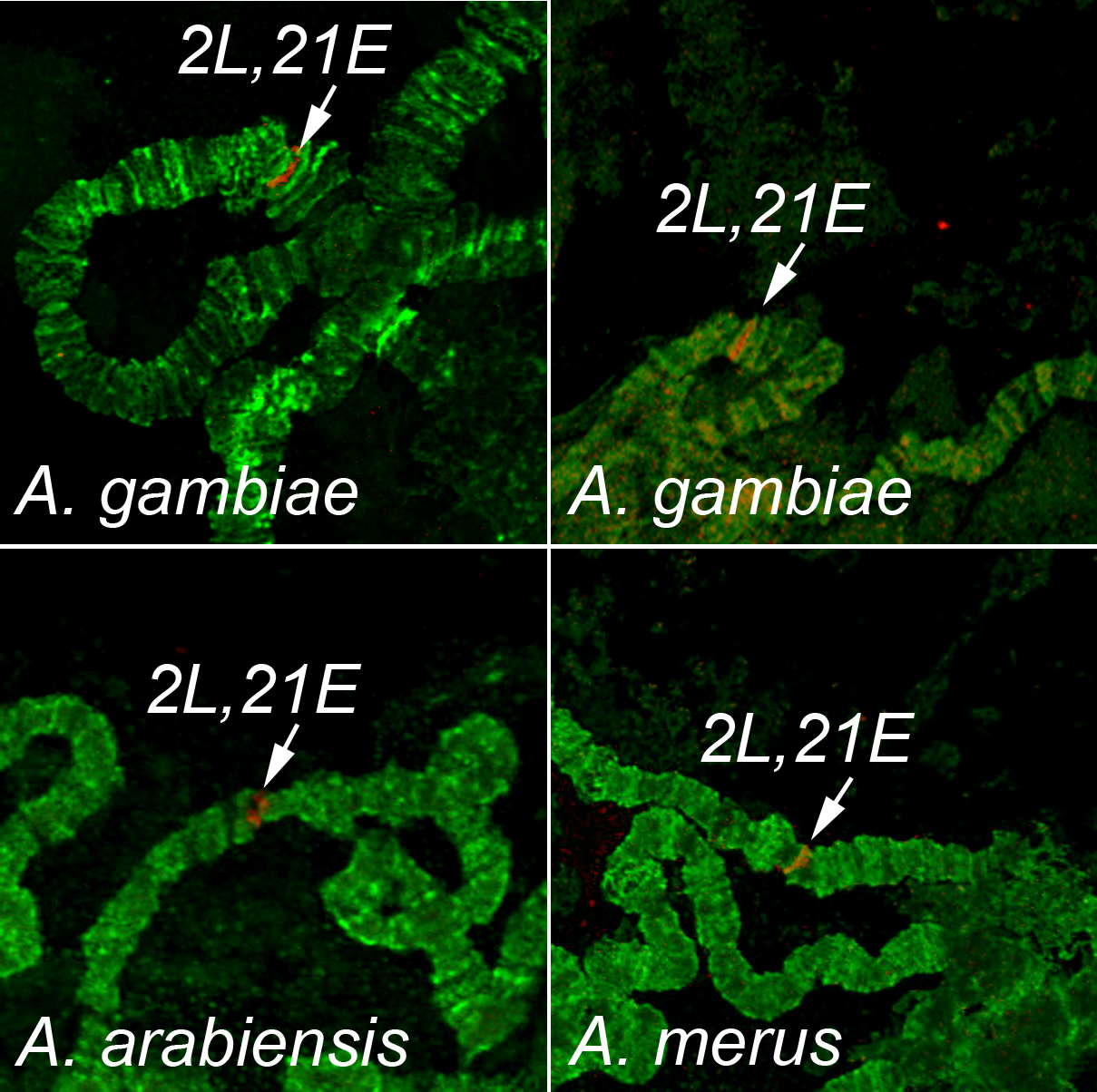

Supplement: Additional file 2 — FISH of AGAP005196 to polytene chromosomes of A. gambiae, A. arabiensis, and A. merus. Top panel: FISH of the AGAP005196 probe encompassing the 3rd intron (left) and the AGAP005196 probe excluding the 3rd intron (right) to polytene chromosomes of A. gambiae. Bottom panel: FISH of the AGAP005196 probe encompassing the 3rd intron to polytene chromosomes of A. arabiensis and A. merus. Arrows indicate the single site of hybridization in the division 21E of the 2L arm. [file 1471-2148-11-72-S2.TIFF]

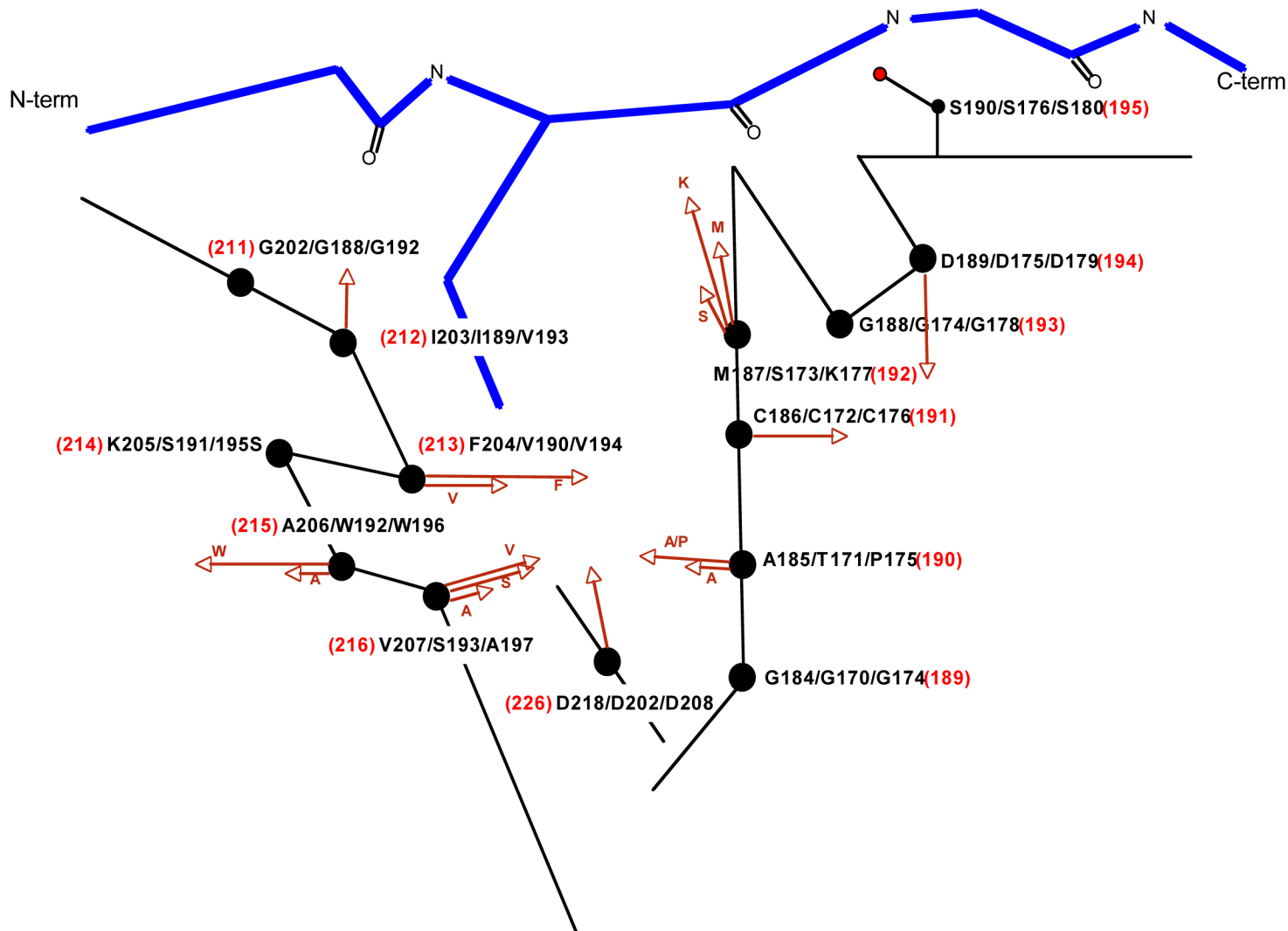

Supplement: Additional file 4 — Scheme of the specificity pocket of the three serine proteases. Residues that contribute to the shape of the pocket are represented with filled circles. Residue type and positions in the AGAP005194, AGAP005195 and AGAP005196 proteases are separated by a "/". Red numbers in brackets indicate the residue position according to the chymotrypsin numbering scheme. Arrows indicate the predicted direction of the residue side chain as deduced by reconstructed models, with the length of the arrow being proportional to the size of the side chain. [file 1471-2148-11-72-S4.PDF]

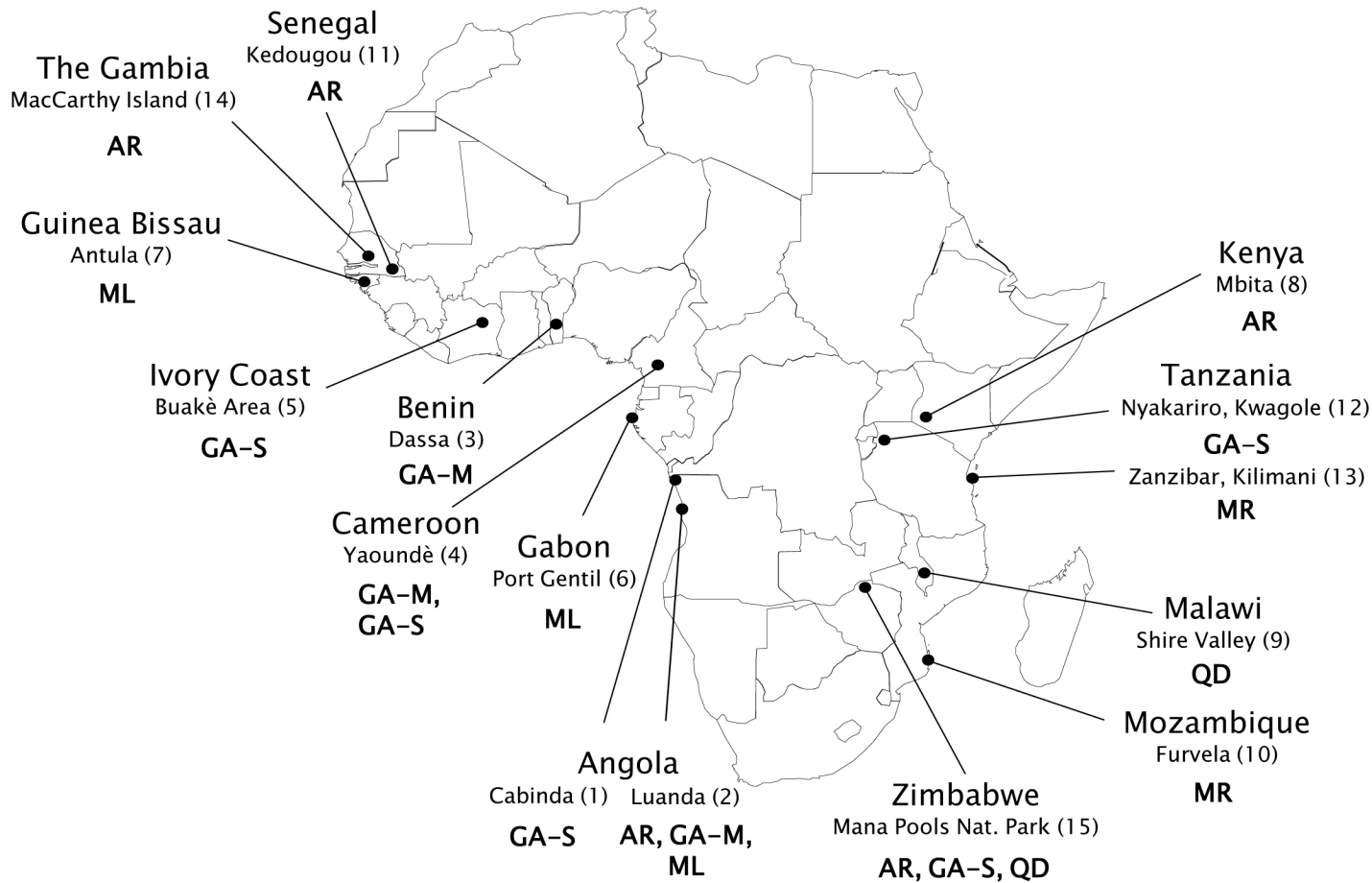

Supplement: Additional file 5 — Map of sampling localities. Numbers in parenteses and species abbreviations after collection sites were used to indicate species and geographic origins of sequence vouchers submitted to GenBank. Sequences from 1 to 8 individuals per species for all genes were obtained from each locality [except for GA-M from Benin (AGAP005194 only), GA-S from Tanzania and AR from The Gambia (AGAP005194 and AGAP005195 only)]. [file 1471-2148-11-72-S5.PDF]
