# Supplementary material for: Molecular evolution of a gene cluster of serine proteases expressed in the Anopheles gambiae female reproductive tract
Source: BMC Evol Biol. 2011 Mar 19;11:72. doi: 10.1186/1471-2148-11-72 (PMC3068966; doi:10.1186/1471-2148-11-72)
Supplement: Additional file 3 — Genetic polymorphisms. Nucleotide polymorphisms of AGAP005194 (= 489 bp), AGAP005195 (= 603 bp), AGAP005196 (= 456 bp) computed using DNAsp ver. 4. [file 1471-2148-11-72-S3.DOC]

**Additional file 3 - Genetic polymorphisms**
