# Supplementary material for: Molecular evolution of a gene cluster of serine proteases expressed in the Anopheles gambiae female reproductive tract
Source: BMC Evol Biol. 2011 Mar 19;11:72. doi: 10.1186/1471-2148-11-72 (PMC3068966; doi:10.1186/1471-2148-11-72)
Supplement: Additional file 6 — Primer table. Sequences of primers used for the amplification of selected portions of female serine protease genes. [file 1471-2148-11-72-S6.DOC]

**Additional file 6 - Primer table**
